# Application of amphiregulin in IVM culture of immature human oocytes and pre-insemination culture for COCs in IVF cycles

**DOI:** 10.3389/fendo.2024.1428147

**Published:** 2024-06-18

**Authors:** Yongqi Fan, Jing Wang, Tingting Ye, Dandan Yang, Qiqi Zhang, Chao Zhang, Bo Yan, Qiushuang Wang, Ding Ding, Beili Chen, Weiwei Zou, Dongmei Ji, Huijuan Zou, Zhiguo Zhang

**Affiliations:** ^1^ Department of Obstetrics and Gynecology, the First Affiliated Hospital of Anhui Medical University, Hefei, Anhui, China; ^2^ National Health Commission (NHC) Key Laboratory of Study on Abnormal Gametes and Reproductive Tract, Anhui Medical University, Hefei, Anhui, China; ^3^ Department of Second Clinical College, Anhui Medical University, Hefei, Anhui, China

**Keywords:** amphiregulin, immature oocytes, *in vitro* fertilization (IVF), *in vitro* maturation (IVM), infertility

## Abstract

**Background:**

Amphiregulin (AR) is a growth factor that resembles the epidermal growth factor (EGF) and serves various functions in different cells. However, no systematic studies or reports on the role of AR in human oocytes have currently been performed or reported. This study aimed to explore the role of AR in human immature oocytes during *in vitro* maturation (IVM) and *in vitro* fertilization (IVF) in achieving better embryonic development and to provide a basis for the development of a pre-insemination culture medium specific for cumulus oocyte complexes (COCs).

**Methods:**

First, we examined the concentration of AR in the follicular fluid (FF) of patients who underwent routine IVF and explored the correlation between AR levels and oocyte maturation and subsequent embryonic development. Second, AR was added to the IVM medium to culture immature oocytes and investigate whether AR could improve the effects of IVM. Finally, we pioneered the use of a fertilization medium supplemented with AR for the pre-insemination culture of COCs to explore whether the involvement of AR can promote the maturation and fertilization of IVF oocytes, as well as subsequent embryonic development.

**Results:**

A total of 609 FF samples were examined, and a positive correlation between AR levels and blastocyst formation was observed. In our IVM study, the development potential and IVM rate of immature oocytes, as well as the fertilization rate of IVM oocytes in the AR-added groups, were ameliorated significantly compared to the control group (All P < 0.05). Only the IVM-50 group had a significantly higher blastocyst formation rate than the control group (P < 0.05). In the final IVF study, the maturation, fertilization, high-quality embryo, blastocyst formation, and high-quality blastocyst rates of the AR-added group were significantly higher than those of the control group (All P < 0.05).

**Conclusion:**

AR levels in the FF positively correlated with blastocyst formation, and AR involvement in pre-insemination cultures of COCs can effectively improve laboratory outcomes in IVF. Furthermore, AR can directly promote the *in vitro* maturation and developmental potential of human immature oocytes at an optimal concentration of 50 ng/ml.

## 1 Introduction

In 1988, amphiregulin (AR), an epidermal growth factor (EGF)-like growth factor, was found to bind only to the EGF receptor (EGFR) and regulate the growth of MCF-7 breast tumor cells in a serum-free medium (1). After binding to EGFR, AR activates a basic cascade of intracellular signaling in the cell cycle (2, 3). AR activation is driven by multiple factors, including cytokines, hormones, and growth factors (4). AR promotes the growth and survival of normal and transformed target cells depending on their concentration and properties (5). AR plays an important role in the development of mammary glands and bone tissues (5, 6).

During the development of mammalian follicles, granulosa cells and oocytes communicate with each other via signaling and chemical communication to regulate meiosis completion in oocytes under gonadotropin stimulation (7, 8). During oocyte growth, the continuous accumulation of cyclin AMP (cAMP) generated by the G protein coupled-receptor (GPR3/12) signaling pathway inhibits the maturation-promoting factor (MPF) activity, leading to oocyte arrest at prophase I of meiosis (8, 9). Once the oocyte is stimulated by luteinizing hormone/human chorionic gonadotropin (LH/HCG) secreted from the ovary, the expression of three EGFR ligands, namely AR, epiregulin (EREG), and betacellulin (BTC), interact with cumulus cells and subsequently activate the EGFR signal in cumulus cells, followed by activating PDE5 to reduce cGMP supply and trigger cAMP hydrolysis, thereby alleviating MPF inhibition and ultimately achieving the goal of promoting cumulus expansion and oocyte maturation (10, 11). Therefore, we speculated that AR plays a crucial role in promoting oocyte maturation. Further systematic research is needed to determine the specific correlation between AR and oocyte maturation or quality.

In this study, we first investigated the correlation between AR levels in the human follicular fluid (FF) of patients who received a routine *in vitro* fertilization (IVF) and oocyte maturation and subsequent embryonic development by detecting the concentrations of AR.

Next, we explored whether AR could directly and effectively promote the *in vitro* maturation (IVM) of immature human oocytes by transferring the collected immature oocytes from controlled ovarian hyperstimulation (COH) cycles into a newly designed IVM medium supplemented with AR for IVM culture. The results showed that the involvement of AR significantly improved the IVM of immature human oocytes.

Finally, based on the above results, we designed another experiment: during IVF, after oocyte retrieval, the cumulus-oocyte complexes (COCs) collected from patients undergoing IVF were transferred into a newly conceived fertilization medium supplemented with AR for 4–6 h of *in vitro* culture, which is a routine culture process before insemination, aiming to further promote cytoplasmic maturation of the retrieved oocytes for subsequent ideal outcomes of fertilization and embryonic development. To date, there is no specialized effective pre-insemination culture medium for COCs. Normally, COCs undergo this culture process in a commercial fertilization medium. This study aimed to explore whether the involvement of AR could effectively induce cytoplasmic maturation of  IVF oocytes, thereby achieving better embryonic development, and provide a basis for the development of a pre-insemination culture medium specific for COCs.

## 2 Materials and methods

### 2.1 Chemicals and reagents

All reagents and chemicals were purchased from Cooper Surgical (USA) unless otherwise noted. See Appendix 1 for more details.

### 2.2 Study design

This study involved three prospective experiments (I, II, and III).

In experiment I, the FF samples of patients undergoing IVF were collected, and AR concentration in the FF was examined by enzyme-linked immunosorbent assay (ELISA). Subsequently, the correlation between AR levels, oocyte maturation, and subsequent embryonic development was systematically analyzed.

In experiment II, the immature oocytes (those at germinal vesicle [GV] or metaphase I [MI] stage) from intracytoplasmic sperm injection (ICSI) cycles were collected. These GV/MI oocytes were divided into four groups of IVM culture: IVM-0 group as the control (in which the IVM medium had no AR) and IVM-50, IVM-100, and IVM-150 as the study groups, with 50 ng/ml, 100 ng/ml, and 150 ng/ml of AR, respectively in the IVM medium. The rates of maturation, fertilization, and embryo development of the four groups were systematically analyzed. It was found that 50 ng/ml was the optimal concentration. Therefore, 50 ng/ml was set as the concentration for subsequent experiments.

**Figure 1:**
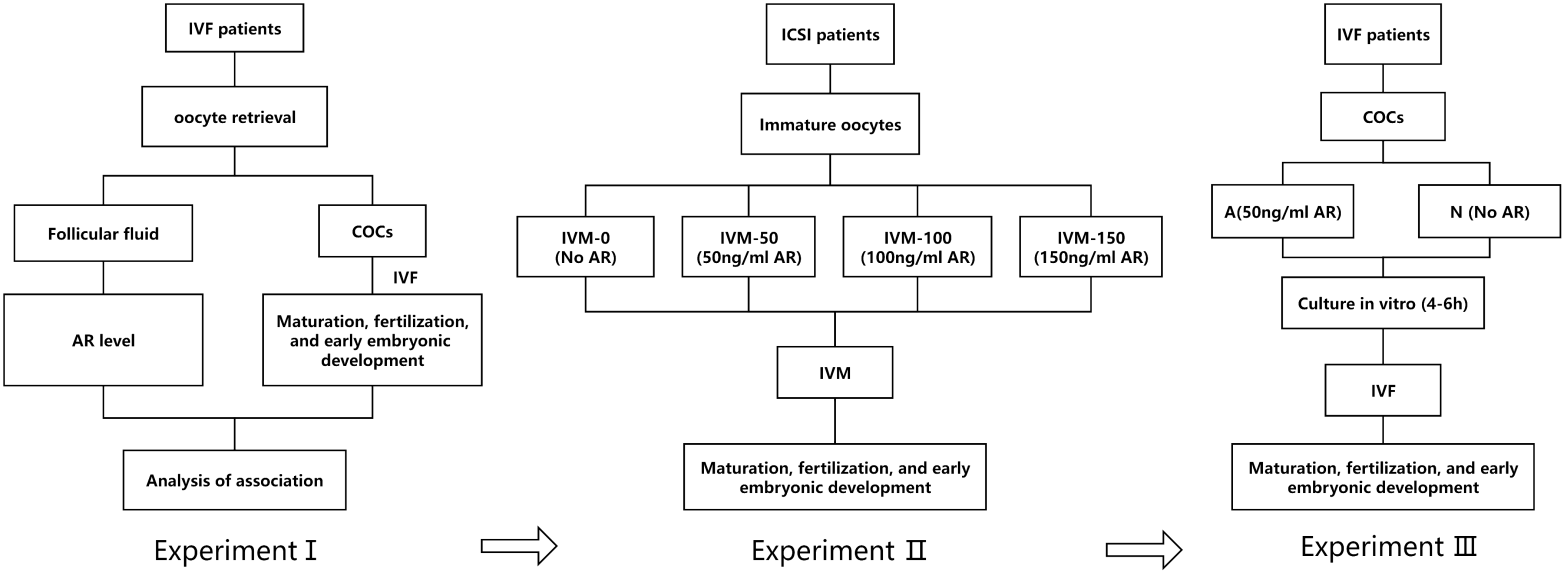
A: Flowchart of the experimental design; I: AR level in the FF of patients; II: Adding AR to IVM; III: AR was added to the insemination pretreatment solution.

Experiment III involved a pre-insemination culture of COCs during IVF. After oocyte retrieval, a certain proportion of the COCs from the same group of patients were placed in a fertilization medium supplemented with 50 ng/ml AR for 4–6 h for *in vitro* culture before insemination. The remaining COCs were cultured in a commercial fertilization medium with no AR, followed by IVF and early embryo culture. The former was the study group (group A), whereas the latter was the control group (group N). The experimental flowchart is shown in **Figure 1**.

### 2.3 Ovarian stimulation regimen, ethics, and informed consent

All patients underwent the conventional ovarian stimulation regimen described in our previous reports (12). This study was approved by the Medical Ethics Committee of the First Affiliated Hospital of Anhui Medical University (No. 20240111) and was initiated and conducted after the patients signed an informed consent form.

### 2.4 FF collection and AR measurement

The FF for this study was obtained from female patients undergoing IVF, aged 20–49 years (median: 32 years), with causes of infertility, including polycystic ovary syndrome (PCOS), chronic salpingitis, and endometriosis. During oocyte retrieval, the second tubule of FF from each patient was collected and subsequently centrifuged at 2000 r/min for 15 **min** to remove cellular structures and debris. After centrifugation, the FF was stored at –20 °C until ELISA, and the concentration of AR in the FF was detected using tetramethylbenzidine as a substrate. The absorbance (OD value) at 450 nm was measured using a 450 series microplate reader, and the AR concentration of each sample was calculated after generating a standard curve using a dilution series of human recombinant protein. The lower limit of detection concentration used for the sample concentration was 0.1 ng/ml.

### 2.5 Immature oocyte collection and IVM

GV/MI oocytes with normal morphology from female ICSI patients (aged ≤ 35 years) were collected and transferred into an IVM medium for 24–32 h of IVM culture, followed by ICSI insemination and 5–6 days of early embryo culture. Our conventional IVM medium, composed of 80% (v/v)TCM199 + 20% (v/v) patient serum + 0.075 IU/ml FSH (follicle stimulating hormone) + 0.5 IU/ml HCG + 0.8 μg/ml estradiol (E2) + 10 μM melatonin and different concentrations of AR added, was used. IVM medium preparation and operational details were similar to those described in our previous reports (13).

### 2.6 Fluorescence staining of AR and EGFR in GV/MI oocytes and granulosa cells

One GV oocyte and one MI oocyte with few granulosa cells were stained using specific antibodies to locate AR and EGFR. First, rabbit anti-amphiregulin antibody and mouse anti-EGFR antibody (the first antibody) were combined with specific antigens on GV/MI oocytes and granulosa cells and the corresponding secondary antibodies with green and red fluorescence, respectively, were applied to bind the first antibody. Finally, fluorescence signals were recorded and analyzed using a confocal microscope (Zeiss LSM 800). See Appendix 1 for more details.

### 2.7 Pre-insemination culture for COCs in IVF cycles

The COCs from IVF patients aged ≤ 35 years were collected. One-third of the COCs from each patient were randomly picked and transferred into a fertilization medium containing 50 ng/ml AR for 4–6 h under *in vitro* culture conditions of 6% CO2, 5% O2, and saturated humidity, whereas the remaining two-thirds’ were cultured in commercial fertilization medium, followed by IVF insemination.

### 2.8 Insemination, early embryo culture, and development assessment

Matured *in vitro* (IVM) or *in vivo* (IVO) oocytes were inseminated via ICSI or routine IVF. After fertilization, normally fertilized oocytes were sequentially transferred into a cleaved embryo and blastocyst *in vitro* media for 2 days culture under conditions of 6% CO2, 5% O2, and saturated humidity. During this period, embryonic development was assessed based on Tomas (14) and Gardner criteria (15). Details of insemination, early embryo culture, and developmental assessment have been described previously (16).

### 2.9 Statistical analysis

Statistical analysis was conducted using GraphPad Prism 8.0 and the statistical package for the Social Sciences 23.0 software. Measurement data were presented as means ± standard deviation, while enumeration data were expressed as percentages. The measurement data were analyzed by two independent sample t-tests between two groups. One-way analysis of variance and Bonferroni test were used to evaluate the statistical significance of differences between three or more groups. The χ2 test was utilized for enumeration data. Spearman test was used for correlation analysis, and P-values < 0.05 were considered statistically significant.

## 3 Results

### 3.1 AR levels in FF correlated with the number of blastocysts formed

**Figure 2:**
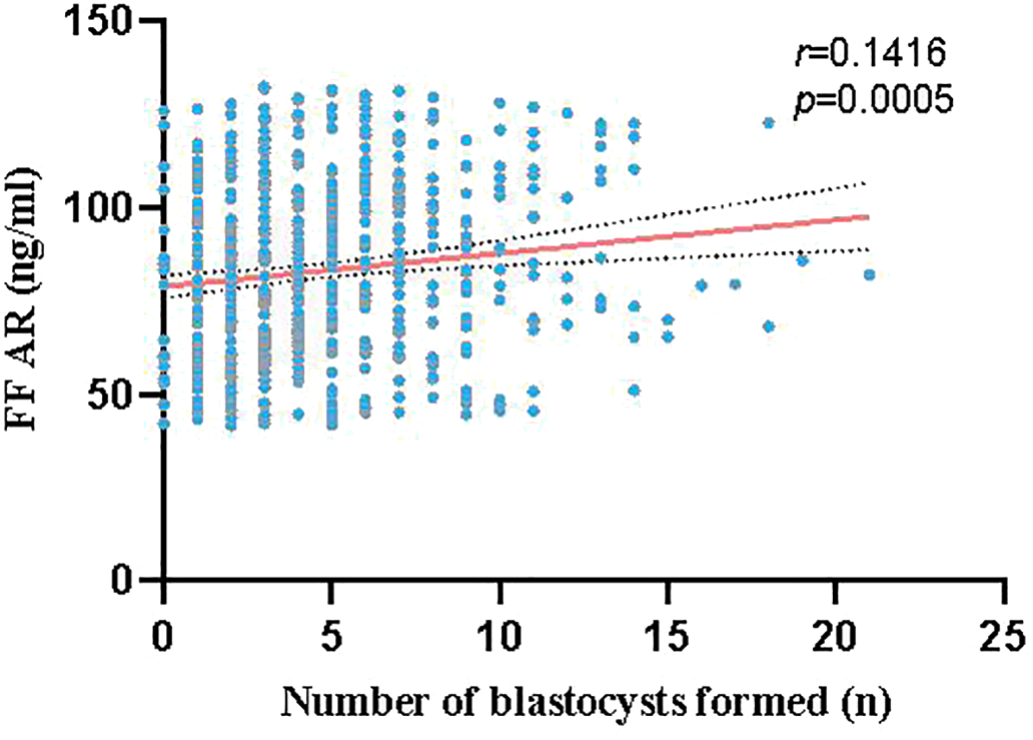
Correlation between AR levels in the FF and number of blastocysts formed (red line represents linear fitting, gray dashed line represents confidence interval): The red line shows a positive correlation trend between the AR levels in the FF and number of blastocysts formed.

A total of 609 couples who underwent IVF and had at least one blastocyst were enrolled. The 609 samples were divided into three groups based on the AR levels in the FF: L group (41.73 ng/ml ≤ AR value ≤ 68.88 ng/ml, n = 203), M group (68.98 ng/ml ≤ AR value ≤ 95.20 ng/ml, n = 203), and H group (95.36 ng/ml ≤ AR value ≤ 133.13 ng/ml, n = 203). AR levels in the FF positively correlated with the number of blastocysts formed, with a positive correlation trend between the two, as shown in **Figure 2**. As presented in **Table 1**, no significant differences were found between the age and FSH, LH, E2, and progesterone (P) levels of the three groups; however, a significant difference was observed between the AR levels of the L, M, and H groups (P < 0.0001). No significant differences were observed between the numbers of oocytes retrieved and the maturation, fertilization, cleavage, and high-quality embryo rates of the three groups. However, blastocyst formation rates differed significantly among these groups (L vs. M: 45.65% vs. 53.63%, P < 0.0001; L vs. H: 45.65% vs. 59.81%, P < 0.0001; and M vs. H: 53.63% vs. 59.81%, P < 0.0001). For high-quality blastocysts rate, only the difference between the L and H groups was statistically significant (42.83% vs. 46.41%, P < 0.0001), and the rate of high-quality blastocysts increased with the increase of AR levels in the FF.

**Table 1:** Basic information and embryonic development of 609 patients.

Index	L	M	H	P1-value (L vs M)	P2-value (L vs H)	P3-value (M vs H)
The number of cycle (n)	203	203	203	-	-	-
Age (years)	31.75 ± 4.791	31.86 ± 4.667	32.33 ± 4.806	0.9699	0.4669	0.6399
FSH (IU/L)	7.006± 2.316	7.466 ± 2.983	7.202± 2.943	0.2790	0.8008	0.6997
LH (IU/L)	6.061± 4.031	6.793± 4.938	5.978± 4.315	0.2958	0.9850	0.2817
E2 (pg/ml)	128.7± 135.1	134.1 ± 96.17	122.6 ± 73.49	0.8970	0.8742	0.6579
P (ng/ml)	2.091± 8.770	1.715± 5.772	2.061 ± 5.535	0.8864	0.9993	0.9174
AR (ng/ml)	56.74± 7.516	81.73 ± 7.644	110.6 ± 10.27	<0.0001*	<0.0001*	<0.0001*
Retrieved oocytes (n)	11.66 ± 7.414	11.51 ± 6.660	11.42 ± 7.679	0.9777	0.9412	0.9909
Rate of maturity (%)	86.62(2040/2355)	86.48(2021/2337)	85.90(2011/2341)	0.8836	0.4976	0.5818
Fertilization rate (%)	79.62(1875/2355)	79.38(1855/2337)	78.73(1843/2341)	0.8370	0.4723	0.5859
Cleavage rate (%)	98.13(1840/1875)	97.20(1803/1855)	97.61(1799/1843)	0.0650	0.3063	0.4695
High-quality embryo rate (%)	67.34(1239/1840)	67.44(1216/1803)	66.20(1191/1799)	0.9718	0.4815	0.4364
Blastocyst formation rate (%)	45.65(840/1840)	53.63(967/1803)	59.81(1076/1799)	<0.0001*	<0.0001*	<0.0001*
High -quality blastocyst rate (%)	42.83(788/1840)	45.81(826/1803)	46.41(835/1799)	0.0717	0.0302*	0.7382

L: 41.73 ng/ml ≤ AR ≤ 68.88 ng/ml; M: 68.98 ng/ml ≤ AR ≤ 95.20 ng/ml; H: 95.36 ng/ml ≤ AR ≤ 133.13 ng/ml; FSH: follicle-stimulating hormone; LH: luteinizing hormone; E2: estradiol; P: progesterone; AR: amphiregulin. Rate of maturity: number of mature oocytes/number of retrieved oocytes. Fertilization rate: number of fertilized oocytes/number of oocytes retrieved. Cleavage rate: number of cleavages/number of fertilizations. High-quality embryo rate: number of high-quality embryos/number of cleavages. Blastocyst formation rate: number of blastocysts formed/number of cleavages. High-quality blastocyst rate: number of high-quality blastocysts/number of cleavages.

### 3.2 Ovarian stimulation regimen had no significant effect on AR levels in FF

These 609 patients were allocated into three groups according to the ovarian stimulation regimen: GnRH-ant (Group A, n = 407), GnRH-a (Group B, n = 161), and early follicular phase long protocol (Group C, n = 41). The effects of the different regimens on AR level in FF were further analyzed. As presented in **Table 2**, no significant differences were observed between the basic indicators (age, FSH, LH, E2, and P) or AR levels among the three groups.

**Table 2:** Effects of different ovarian stimulation regimens on AR concentrations.

Index	Group A	Group B	Group C	P1-value (A vs B)	P2-value (A vs C)	P3-value (Bvs C)
The number of cycle(n)	407	161	41	-	-	-
Age (years)	31.67 ± 4.506	32.20 ± 5.370	32.68 ± 4.373	0.4443	0.3923	0.8329
FSH	7.196 ± 2.704	7.084 ± 2.738	7.863 ± 2.937	0.9137	0.3641	0.2970
LH(mIU/ml)	6.376 ± 4.709	5.621 ± 3.323	6.673 ± 4.040	0.1996	0.9231	0.4140
E2(pg/ml)	160.1 ± 292.8	148.7 ± 270.8	133.7 ± 56.80	0.9137	0.8611	0.9584
P(ng/ml)	1.508 ± 3.152	2.068 ± 5.170	1.022 ± 1.083	0.3143	0.7544	0.3194
AR(ng/ml)	82.02 ± 23.19	85.09 ± 24.57	86.63 ± 24.12	0.3453	0.4603	0.9265

### 3.3 AR in IVM culture improved the development potential of immature oocytes

**Figure 3:**
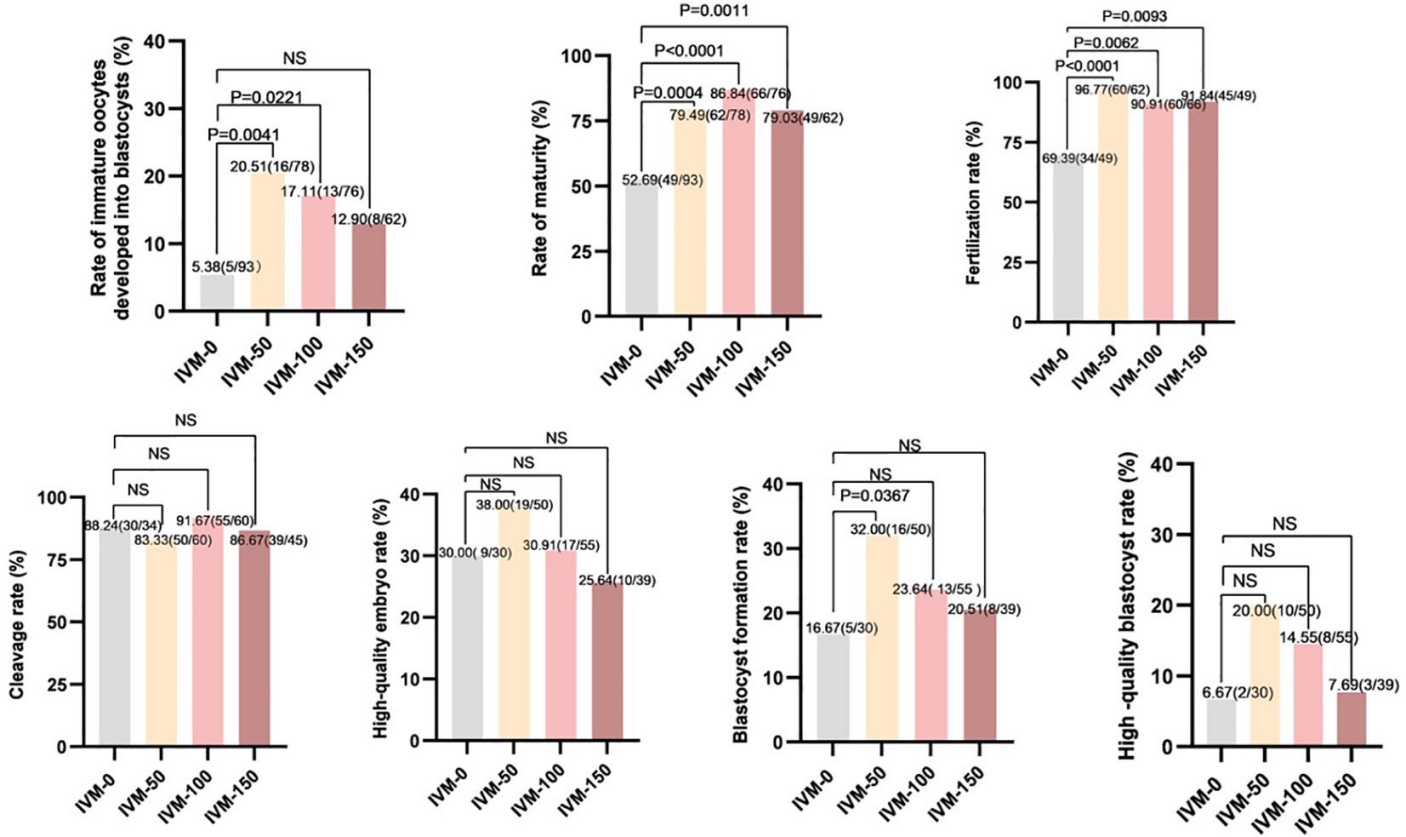
Comparison between embryo development and different concentrations of AR during IVM. IVM-0: No AR added; IVM-50: 50 ng/ml AR was added; IVM-100: 100 ng/ml AR was added; IVM-150: 150 ng/ml AR was added; Rate of immature oocytes developed into blastocysts: number of blastocysts formed/total number of oocytes retrieved: Rate of maturity: number of mature oocytes/number of retrieved oocytes. Fertilization rate: number of fertilized oocytes/number of oocytes retrieved. Cleavage rate: number of cleavages/number of fertilizations. High-quality embryo rate: number of high-quality embryos/number of cleavages. Blastocyst formation rate: number of blastocysts formed/number of cleavages. High-quality blastocyst rate: number of high-quality blastocysts/number of cleavages.

A total of 309 immature oocytes were retrieved from patients who underwent ICSI in COH cycles, including 231 GV (n = 231) and 78 MI (n = 78) oocytes. As shown in **Figure 3** (IVM-0), five oocytes of the 93 immature oocytes in the control group developed to the blastocyst stage (5.38%), whereas thirty-seven oocytes (of the 216 immature oocytes) developed to the blastocyst stage (17.13%) in the AR-supplemented groups (IVM-50, -100, and -150). Among them, the IVM-50 group had the highest proportion (20.51%) of immature oocytes that developed to the blastocyst stage. Compared to the IVM-0 group, the developmental potential of immature oocytes in the AR-supplemented groups was ameliorated, especially in the IVM-50 (20.51% vs. 5.38%, P < 0.01) and IVM-100 groups (17.11% vs. 5.38%, P < 0.05). Next, we conducted further analyses on IVM, fertilization, and early embryo development rates. The IVM and fertilization rates of each AR-supplemented group were significantly higher than those of the control group (All P < 0.05); however, regarding the cleavage, high-quality embryo, and high-quality blastocyst rates, each AR-supplemented group was comparable to the control group (**Figure 3**). Furthermore, regarding the blastocyst formation rate, only the IVM-50 group had a significantly higher blastocyst formation rate than the control group (32.00% vs. 16.67%, P < 0.05), while the other groups were comparable to the control group (**Figure 3**). These results indicate that AR, as an additive to IVM medium, can effectively improve the developmental potential of immature human oocytes, and 50 ng/ml is the optimal concentration.

### 3.4 AR and EGFR are present on GV/MI oocytes and their granulosa cells.

**Figure 4:**
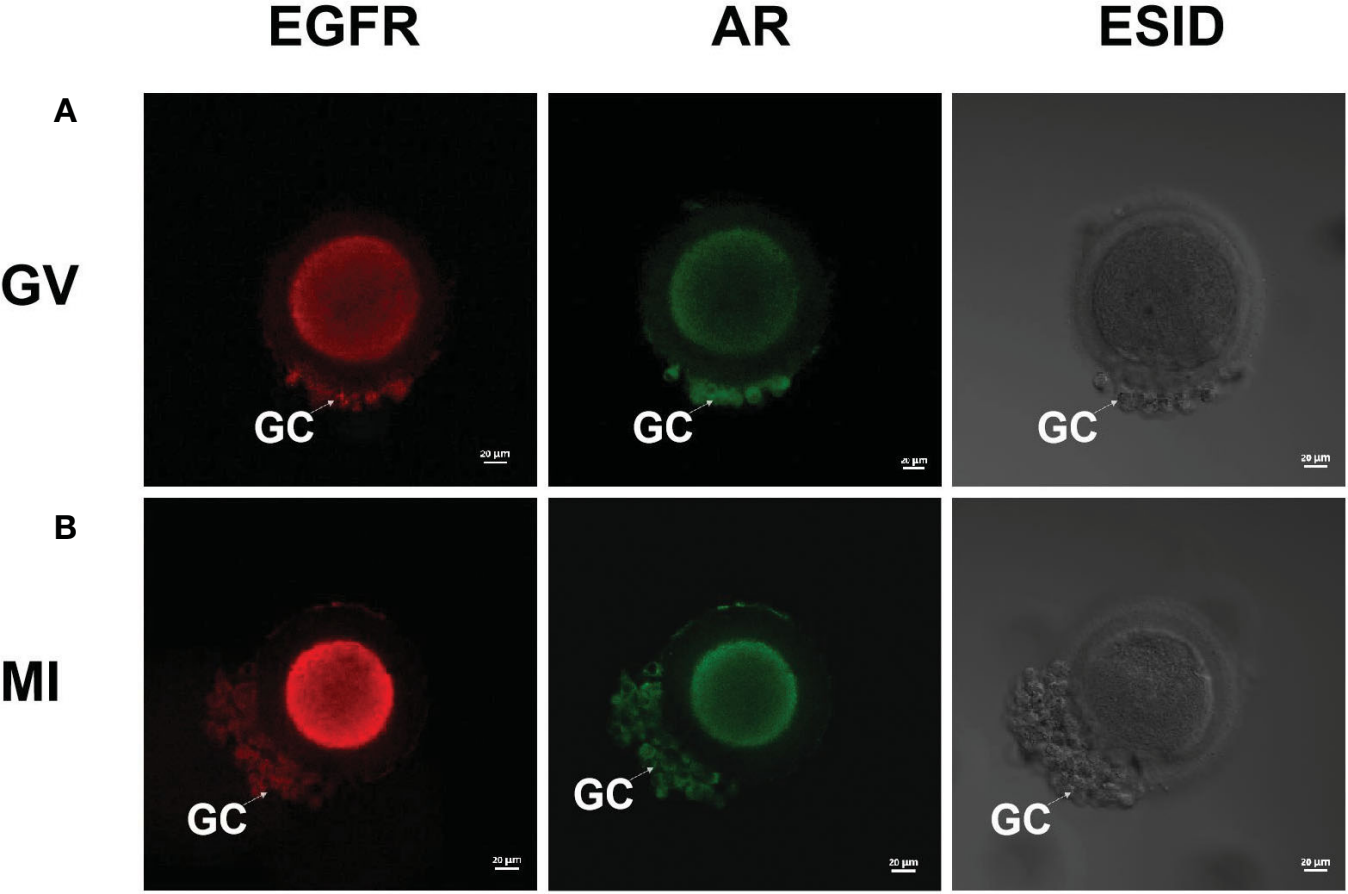
Fluorescence staining of AR and EGFR in GV/MI oocytes and granulosa cells (Red and green color represented the signal of EGFR and AR, respectively). **(A)** showed the presence of AR and EGFR in the GV oocyte and granulosa cells, and **(B)** showed that in the MI oocyte and its granulosa cells. GC, granulosa cells. Bar = 20 μm.

As shown in **Figure 4**, red and green fluorescence signals were observed on the plasma membranes of the two oocytes and their granulosa cells; however, no signals were observed on the zona pellucida. This means that EGFR exists not only on the granulosa cells but also on the plasma membrane of immature human oocytes.

### 3.5 AR can improve the laboratory outcomes of IVF cycles

Sixteen patients who underwent IVF participated in experiment III, and 325 COCs were obtained, of which 113 were assigned to group A and 212 to group N for pre-insemination culture. As presented in **Table 3**, group A had a significantly higher maturation rate (98.23% vs. 84.91%, P < 0.0001), fertilization rate (94.69% vs. 75.00%, P < 0.0001), high-quality embryo rate (82.08% vs. 69.43%, P = 0.0217), blastocyst formation rate (66.04% vs. 45.22%, P = 0.0010), and high-quality blastocyst rate (100% vs. 71.83%, P < 0.0001) than group N. However, no significant differences were observed between the two groups regarding fertilization and cleavage rates. These results indicate that the application of AR in the pre-fertilization culture of COCs can effectively improve the laboratory outcomes of IVF cycles.

**Table 3:** Comparison of embryo development between two groups in pre-fertilization medium.

Index	A	N	P-value
Number of cases (n)	16	16	–
No.of oocytes retrieved (%)	113	212	–
Rate of maturity (%)	98.23(111/113)	84.91 (180/212)	<0.0001*
Rate of 2PN (%)	66.37 (75/113)	60.85 (129/212)	0.3381
Fertilization rate (%)	94.69 (107/113)	75.00 (159/212)	<0.0001*
Cleavage rate (%)	99.07 (106/107)	98.74 (157/159)	>0.9999
High-quality embryo rate (%)	82.08 (87/106)	69.43 (109/157)	0.0217*
High-quality blastocyst rate (%)	66.04 (70/106)	45.22 (71/157)	0.0010*
Proportion of high-quality blastocysts (%)	100 (70/70)	71.83 (51/71)	<0.0001*

A: The pre-fertilization treatment solution was supplemented with 50 ng/ml AR. N: No AR was added to the pre-fertilization treatment solution. Rate of maturity: number of mature oocytes/number of retrieved oocytes; fertilization rate: number of fertilized oocytes number of oocytes retrieved; rate of 2PN: number of 2PN/number of mature oocytes. Cleavage rate: number of cleavages/number of fertilizations; high-quality embryo rate: number of high-quality embryos/number of cleavages. High-quality blastocyst rate: number of high-quality blastocysts/number of cleavages. The proportion of high-quality blastocysts: number of high-quality blastocysts/total number of blastocysts.

## 4 Discussion

A previous study showed that AR is the most abundant EGF-like growth factor in the FF of patients who received HCG stimulation (17). EGF-like growth factors are paracrine mediators that transmit LH signals throughout the follicles (18). The appearance of the LH peak plays a key role in promoting ovulation, triggering a series of events during follicular development, including the recovery of oocyte meiosis, cumulus expansion, and rupture of the follicle wall; it also plays a role in the extrusion of COCs (19). The surge in LH levels synergistically promotes the activation of cGMP phosphodiesterase PDE5 in granulosa cells, leading to a decrease in cGMP supply to oocytes (10). Low levels of intracellular cGMP induce cAMP hydrolysis in oocytes (20). cAMP hydrolysis reduces the ability of cyclin-dependent kinase 1 to induce PKA-dependent phosphorylation in oocytes, thereby reducing the inhibition of meiosis-promoting factors (MPF) and restoring meiosis (21). EGFR ligands (AR and EREG) interfere with the flow of meiosis inhibitors, cAMP and cGMP, to oocytes through the phosphorylation of connexins, leading to a sustained reduction in cGMP concentration throughout the follicle (22). It can be inferred that AR can further reduce cGMP in oocytes based on LH peak stimulation, intensify cAMP hydrolysis and MPF activation, induce the recovery of oocyte meiosis, and promote cytoplasmic maturation of human oocytes, thus having a positive significance for subsequent embryo development. In 2011, AR increased blastocyst rate and blastomere number in porcine oocytes (23); however, current reports on the correlation between AR and human oocytes are insufficient. In our study, a total of 609 FF samples were examined. The results showed that AR levels in FF positively correlated with the number of blastocysts formed, which means that it can provide predictions for the laboratory outcomes of patients undergoing IVF in clinical practice. We found no correlation between the different ovulation stimulation regimens and AR levels. Therefore, clinicians should develop personalized ovulation induction protocols based on the patient’s condition during IVF.

IVM technology, an important backup for IVF and ICSI technology, has a positive effect on the treatment of some special patients, such as those with PCOS and oocyte developmental disorders. The pregnancy rate of IVM oocytes was much lower than that of IVO oocytes, indicating that improvement of IVM technology is urgently needed (24). Our previous study focused mainly on the IVM of immature human oocytes from the COH cycle. Previous studies have shown that immature oocytes can be transformed through IVM technology. In our previous studies, we developed a novel IVM medium based on melatonin addition and applied it in clinical practice, ultimately achieving satisfactory results (13). However, limitations, such as poor stability of IVM effects and a low rate of blastocyst formation in IVM oocytes, have been observed. Therefore, continual optimization of our IVM technology system remains the focus of our research. AR plays a positive role in the IVM culture of mice (25), pigs (23), cows (26), and monkeys (27) and is considered a marker of cytoplasmic maturation in oocytes (9). This could improve the effects of IVM on immature human oocytes during routine IVM therapy (28, 29). Therefore, in this study, based on our previous results, AR was added to our self-developed IVM medium for the culture of immature human oocytes in COH cycles, followed by ICSI insemination for IVM oocytes and early embryo culture. We discovered that the development potential and IVM and fertilization rates of immature oocytes in the AR-added groups were ameliorated, especially in the IVM-50 group. Moreover, the blastocyst formation rate was significantly higher in the IVM-50 group than in the control group. These results indicate that AR, as an additive to IVM medium, can effectively enhance the developmental potential of immature human oocytes in COH cycles. A correlation between AR concentration and immature oocyte development was observed, with 50 ng/ml being the optimal AR concentration for action.

Amanda C. et al. demonstrated the efficacy of EGFR inhibitors in inhibiting mitogenactivated protein kinase (MAPK) 3/1 pathway in defolliculated zebrafish oocytes, reducing spontaneous maturation of defolliculated zebrafish oocytes (30). In this study, IVM culture of GV/MI oocytes was conducted in the absence of granulosa cells, and the involvement of AR was found to significantly increase the IVM rate and development potential. According to previous studies, the expression of EGF and EGFR in human granulosa cells began only in the sinus phase, whereas in pre-antral follicles, the expression of EGF and EGF receptors was limited to oocytes (31). A study in goats found that EGFR transcripts were present in follicular cells, meiotic competent oocytes, and noncompetent oocytes (32). Ríos et al., in their study of bovine oocytes, found that the pro-maturation effect of EGF was evident in degranulated oocytes, indicating that EGF acts directly, or at least partially, on oocytes (33). A study on mice showed that the zona pellucida allows molecular substances, up to 150 kDa, to pass through (34, 35). Therefore, we speculated that the human zona pellucida also has this characteristic. In this study, we performed fluorescence staining for AR and its receptor (EGFR) in GV/MI oocytes and granulosa cells. The results revealed the absence of AR and its receptor within the zona pellucida, while a significant distribution of receptors was observed in the granulosa cells and plasma membrane. Therefore, we speculated that AR may interact with receptors in the plasma membrane through channels in the zona pellucida to promote the IVM and development potential of immature human oocytes.

Normally, during IVF, COCs are incubated in a commercial fertilization medium for 4–6 h of pre-insemination culture after oocyte retrieval. This process is known as the IVM culture of the oocyte cytoplasm. If the culture time is too short or too long, it will affect the maturation of the oocyte cytoplasm, ultimately affecting the fertilization rate of oocytes and the quality of subsequently formed embryos. Currently, there is no specialized effective medium for the pre-insemination culture of COCs. Based on the results of experiments I and II, we added 50 ng/ml AR to the fertilization medium for the pre-insemination culture of COCs. The involvement of AR in the pre-insemination culture was found to significantly ameliorate the rates of maturation, fertilization, high-quality embryo, blastocyst formation, and high-quality blastocysts, which indicates that the addition of AR significantly promotes the maturation of oocyte cytoplasm, thereby improving the overall quality of oocytes and ultimately enhancing the laboratory results of patients undergoing IVF. Concurrently, the results also provided a thought for developing a professionally effective medium for the pre-insemination culture of COCs. However, there are some limitations to the study. First of all, because in the process of oocyte retrieval for patients, we need to complete the oocyte retrieval process as quickly as possible, we only obtain FF from a single follicle of each patient. Therefore, the AR concentration may differ from that of the total FF. We cannot rule out the possibility that this may have influenced the results we concluded that AR concentration was positively correlated with blastocyst formation numbers. Second, because FF is not obtained from normal, fertile women, there may be some differences in the physiological maturity of each patient’s follicles.

## 5 Conclusion

In short, it can be concluded that AR levels in FF are positively correlated with blastocyst formation, and AR involvement in the pre-insemination culture of COCs can effectively improve the laboratory outcomes of IVF. Furthermore, AR can directly act on oocytes to promote their *in vitro* maturation and developmental potential at a 50 ng/ml optimal concentration of action.

## Data availability statement

The original contributions presented in the study are included in the article/Supplementary Material. Further inquiries can be directed to the corresponding authors.

## Author contributions

YF: Writing – original draft. JW: Data curation, Software, Writing – review & editing. TY: Formal Analysis, Writing – original draft. DY: Software, Writing – original draft. QZ: Methodology, Writing – review & editing. CZ: Validation, Writing – original draft. BY: Methodology, Writing – review & editing. QW: Supervision, Writing – original draft. DD: Software, Writing – original draft. BC: Project administration, Writing – original draft. WZ: Validation, Writing – review & editing. DJ: Project administration, Writing – original draft. HZ: Resources, Writing – review & editing. ZZ: Funding acquisition, Resources, Writing – review & editing.

## Appendix 1

**Source of reagents**

rhAmphiregulin (R&D Systems)

Alexa Fluor 488-labeled goat anti-rabbit IgG, (Shanghai Haoqi Biotechnology Co., LTD.)

Alexa Fluor 594-labeled goat anti-mouse IgG (H+L) (Shanghai Haoqi Biotechnology Co., LTD.)

Recombinant Anti-EGFR antibody [13/EGFR] ab289889 (Abcam; UK)

Anti-Amphiregulin antibody ab234750 (Abcam; UK)

ELISA kit (Human Amphiregulin (AR) ELISA Kit (MEIMIAN; Jiangsu Meimian Industrial Co., Ltd)

450 series microplate reader. (Sunrise automatic microplate reader F50; Switzerlan)

Pasteur straws Sigma Inc

ICSI injection needles Sunlight Medical, USA

Holding fixed needles Sunlight Medical, USA

Petri dishes Nunc, Denmark

Operating dish American Life Sciences Inc

Frozen carrier rod Kitazato Co., Japan

Inverted microscope Olympus Corporation, Japan

Laser confocal microscopy Zeiss, Germany

Microoperating system Nikon, Japan

1×PBS buffer Irvine Scientific, USA

4% tissue cell fixative Wuhan Moore Biotechnology Co., LTD

Recombinant human follicle stimulating hormone (r-hFSH) Merck Serono, Switzerland

Human chorionic gonadotropin (hCG) Merck Serono, Switzerland

17β-estradiol (17β-estradiol) Sigma Corporation, USA

Hyaluronidase VitroLife, Sweden

Paraffin culture oil VitroLife, Sweden

**Steps for fluorescent staining of AR and EGFR in GV/MI oocytes and granulosa cells**

Experimental procedures:

1. GV-MI eggs were washed 3 times in preheated PBS buffer.
2. After 4% tissue cell fixative was fixed in the dark for 1 hour, the cells were washed 3 times with PBST buffer, 3 minutes each time.
3. Permeabilize with 5% saponin at room temperature for 30 minutes, and then wash with PBST 3 times, 3 minutes each time.
4. GV-MI eggs were blocked in bovine serum for 1 hour and washed with PBST 3 times for 3 minutes each time.
5. GV-MI eggs were transferred to diluted primary antibody and incubated overnight at 4°C in the dark.
6. The eggs were washed 3 times with PBST for 3 minutes each time.
7. GV-MI eggs were transferred to diluted secondary antibody and incubated at room temperature in the dark for 1 hour.
8. The eggs were washed 3 times with PBST for 3 minutes each time.
9. The eggs were transferred to microdroplets in glass dishes and observed under a laser confocal microscope and images were collected.
